# A feasibility study of an artificial intelligence based decision support system for personalised housing adaptations and assistive technology

**DOI:** 10.1007/s44163-026-01130-5

**Published:** 2026-04-11

**Authors:** Divya Saleela, Adekunle S. Oyegoke, Jamiu A. Dauda, Saheed O. Ajayi

**Affiliations:** 1 Clinical and Experimental Sciences, Faculty of Medicine, University of Southampton, Southampton, UK; 2 School of Built Environment, Engineering and Computing, Leeds Beckett University, Leeds, UK

**Keywords:** Housing adaptations, Decision support system, Inclusive design, Assistive technology, Disease and symptoms

## Abstract

This feasibility study explores the development of the HomeAI-Enable system, an AI-driven decision support platform designed to provide personalised housing adaptations and assistive technology recommendations for older adults and individuals with disabilities. The study follows a constructive research approach including focus groups and brainstorming exercises in addition to development research techniques. Three different datasets comprising one, five, and over 500 diseases sourced from reputable databases such as the NHS and Mayo Clinic were used. The datasets include symptoms, diseases and associated housing adaptation and assistive technologies for the identified symptoms and diseases. The system integrates these datasets to offer personalised solutions based on symptoms, disease, body functions, and environmental factors, using advanced techniques like fuzzy matching, natural language processing, and advanced machine learning models. The feasibility study involves the assessment of multiple AI models to determine their effectiveness in providing tailored recommendations for assistive technology and housing adaptations. A proof of concept was developed, and early technical evaluation demonstrates the viability of the proposed architecture and recommendation workflow for housing adaptation decision support. This study evaluates the technical feasibility of the system at a foundational stage within a broader research programme, with clinical validation involving occupational therapists and end users planned for subsequent phases.

**Supplementary Information:**

The online version contains supplementary material available at 10.1007/s44163-026-01130-5.

## Introduction

The demand for housing adaptation for older adults and disabled individuals is growing due to population ageing and the increasing prevalence of disabilities with age [32]. Massive delays in housing adaptations for the elderly and disabled people are putting them at risk of being forced into residential care when most of them would like to live independently at home. About 16 million people, 24% of UK population have a disability in 2021/22, over 1.6 million people had complex disabilities, and 57% more adults were expected to require care in 2038 compared to 2018 [36]. The increasing demand leads to long waiting time that can be over 573 days/19 months [47] when statutorily, assessment and approval should not take more than 6 months [19].

The leading causes of delay can be broadly categorised under the decision-making process in assessment, assistive technology, and design customisation for people with complex disabilities. The Royal College of Occupational Therapists (RCOT) acknowledged these problems in its reports, guiding principles for the assessment and provision of equipment [33], and adaptations without delay [34]. Waiting for a social care assessment is cited by RCOT [34] as a key factor contributing to delays in the delivery of adaptations. This is because the need for an adaptation has been defined by the solution’s type or cost rather than the situation’s complexity. This prevents occupational therapists from concentrating their specialist skills on working with individuals whose circumstances are most complex and where they would have the greatest impact on the individual’s health and wellbeing. This indicates a need to enhance the complex housing adaptation process to reduce pressure on the OT, peoples in care homes and the National Health Service (NHS) in the UK There is recognition that the sooner housing adaptations are made, the greater the preventative benefits [47], e.g. reducing the risk of falls and associated costs. Hip fractures have adverse effects on daily living activities, account for around £2 billion of fragility fractures costs and increase one-year mortality between 18 and 33% in the UK [31].

This study presents a feasibility assessment conducted prior to development of an AI-Driven Decision Support System for Personalised Housing Adaptations and Assistive Technology (HomeAI-Enable). HomeAI-Enable system is developed to improve how housing adaptations and assistive technologies are recommended for older adults and people with disabilities. This feasibility study explored a range of smart and practical methods that use Artificial Intelligence (AI), Machine Learning (ML), Natural language Processing (NLP), Fuzzy and interactive tools to speed up the process of decision making by recommending suitable housing adaptation and assistive technologies for different types of diseases and symptoms. Each method looked at the problem from a different angle, and together they offer a clear path for building a system that is helpful, efficient, and easy to use. The HomeAI-Enable system is designed to match diseases and symptoms with corresponding housing adaptation and assistive technology needs. It comprises one integrated expert-AI decision support system/platform with three modules:

1. Housing Adaptation Needs Assessment Module—Algorithms to prioritise critical housing needs based on health profiles and care/mobility status requirements.
2. The Housing Design Customisation Module incorporates universal design principles, standard technical specifications, and a space standards matrix to ensure accessibility, safety, and comfort.
3. Housing Smart-Devices/Assistive Technology Module identifies and customises assistive technologies aligned with design features and individual needs with evaluative AI to enhance usability and compatibility.

The remainder of this paper is organised as follows. Section 2 reviews related work on AI-driven decision support systems, housing adaptations, and assistive technologies. Section 3 describes the datasets and methodological framework used in this feasibility study, including dataset construction and mapping strategies. Section 4 presents the system design, development, application, and usability of the proposed HomeAI-Enable framework. Section 5 reports the experimental evaluation across the three datasets, highlighting the progression from rule-based models to symptom-driven and LLM-based approaches, which directly led to the development of the final best-performing multitiered model. Finally, Sect. 6 concludes the paper and outlines directions for future enhancement of the proposed system.

## Literature survey

This section reviews the current research and developments relevant to housing adaptations, focusing on the challenges and delays in implementation, common diseases and associated symptoms affecting users, design customisation approaches, and the role of assistive technology in enhancing living environments.

### Delay in housing adaptation

Delays in the implementation of housing adaptations significantly affect older adults and individuals with disabilities, compromising safety and reducing autonomy. Common causes include fragmented interagency working, assessment backlogs, and regional inconsistencies in processing Disabled Facilities Grants (DFGs) [17, 18, 26]. Delay in timely housing adaptation intervention can delay the onset of disability and help maintain independence for a longer time [45]. Social stigma [2], emotional and psychological distress [41], financial barriers [1, 47] and weak policy integration [17, 18] further complicate access. According to Wittenberg et al. [42] and Bottery et al. [5], underfunding in adaptation services results in long-term cost inefficiencies. Carnemolla and Bridge [7] argue that these delays reflect broader ethical and economic shortcomings in care delivery.

According to the RCOT [33, 34], health and social care policy in the UK supports a ‘person-centred’ or ‘citizen-centred’ approach through self-assessment and identification of needs for minor adaptations. Minor adaptations support the prevention agenda, to reduce falls, delay admission to hospital, prevent readmission, etc. However, there continue to be delays for people who can self-direct their care and wish to adapt their homes in straightforward cases [33, 34]. To address these challenges, the HomeAI-Enable system is proposed. The system will facilitate timely assessment by linking diseases and symptoms to appropriate home adaptations and assistive technologies.

### Disease and symptoms

Chronic illnesses such as dementia, arthritis and respiratory conditions increase reliance on functional and adaptable home environments. Sixsmith et al. [37] found that environmental barriers in housing are associated with declines in physical functioning and autonomy among older adults, especially when adaptations are delayed. Inaccessible housing layouts often accelerate frailty and dependence due to reduced mobility and safety. In addition, inadequate housing conditions, including damp, poor insulation and lack of ventilation, have been shown to exacerbate respiratory conditions and increase the risk of hospitalisation, particularly in older populations [38]. These findings underline the role of housing as a health determinant, reinforcing calls for integrating adaptation services into long-term disease management. Sixsmith et al. [37] stressed the importance of psychological safety within the home, highlighting how inaccessible layouts contribute to social withdrawal and reduced emotional well-being. Imrie [20] and the Centre for Ageing Better [9] advocate for preventive action through early housing adaptation, positioning the home as a core aspect of long-term condition management.

### Design customisation

Design customisation plays a crucial role in determining the usability and long-term success of housing adaptations. Imrie [20] argued that standardised solutions often fail to meet the complex needs of individuals, calling instead for inclusive, co-designed approaches. Customised modifications not only enhance functionality but also empower users and promote psychological ownership of the space. Organisations such as Habinteg Housing Association (2024) have promoted flexible housing standards based on Building Regulations (Part M) M4(2) and M4(3), which ensure long-term adaptability. Specifications and technical guidance also play a key role in delivering accessible and inclusive housing that is flexible and adaptable to the changing needs [15]. Foundations UK [13] promotes delivering high-quality, bespoke adaptations that balance accessibility with aesthetics in the local authorities in the UK. Heywood [17, 18] and Adams and Hodges [3] also provide evidence that personalised design contributes to cost effectiveness and improved satisfaction among users.

### Assistive technology

Assistive technology (AT) has emerged as a key enabler of independence for older people and individuals with disabilities. Torres et al. [39] demonstrated that smart sensors, fall alerts, and remote monitoring systems can help reduce dependence on formal care services when properly integrated into home environments. Department of Health and Social Care [12] prioritised investment in AT as part of its health innovation strategy. Despite its promise, barriers to adoption persist partly due to a lack of awareness and access to information. Woolham and Benton [43] found low uptake due to a lack of follow-up support and training. Greenhalgh et al. [14] argued that emotional acceptance and intuitive use are as important as technical effectiveness. These findings highlight the importance of person-centred, rather than tech-centred, deployment strategies.

neurologists.

### Project needs requirements analysis

The project is underpinned by the WHO framework on the International Classification of Functioning, Disability and Health (ICF) [44]. The WHO framework integrates the model of health, daily activities participation functions and the role of environmental factors. Functioning denotes “the positive or neutral aspects of the interaction between a person’s health condition(s) and that individual’s contextual factors (environmental and personal factors)”. Disability denotes “the negative aspects of the interaction between a person’s health condition(s) and that individual’s contextual factors (environmental and personal factors)”. The WHO framework is used because it provides the scientific basis, establishes a common language and provides a systematic coding scheme.

According to the WHO [44], disability has three dimensions: Impairment, activity limitation, and participation restrictions. Therefore, the CDC [8] defines disability as any condition of the body or mind (impairment) that makes it more difficult for the person with the condition to do certain activities (activity limitation) and interact with the world around them (participation restrictions). The disability can affect vision, movement, thinking, remembering, learning, communicating, hearing, mental health and social relationships. The ICF document integrates the three models of health and health-related issues, which comprise impairments, the activities of people/individuals and their participation/involvement in all areas of life, and the environmental factors which affect their experiences.

The WHO [44] framework is analysed through content analysis/document analysis to map out areas that can be used to develop the HomeAI-Enable project framework based on these three basic principles.

- The medical aspect focuses on structural and functional impairments but emphasises the physiological functions of body systems. Looking at it from a person’s body structure or function, or mental functioning, such as a significant deviation or loss.
- The activities and participation limitations deal with the difficulties an individual may have in executing activities. (i) Activities in terms of execution of a task or action by an individual, such as difficulty seeing, hearing, walking, or problem-solving; and (ii) the participation limitations in all areas of life, such as working, engaging in social and recreational activities, etc.
- Environmental factors in the form of barriers to or facilitators of the person’s functioning. It covers the physical, social and attitudinal environment in which people live and conduct their lives. This broadly covers, for instance, products and technology for personal use in daily living and the design, construction and building products and technology of buildings.

Additionally, the ISO 9999:2022 classification document on assistive products for persons with disability was used to add assistive technology to the framework. The ISO 9999:2022 classification system divides products into 12 main categories, each representing a general type of assistive product. Nine of the categories apply to the study because they focus on personal care, comfort, security and communication [22].

**Fig. 1:**
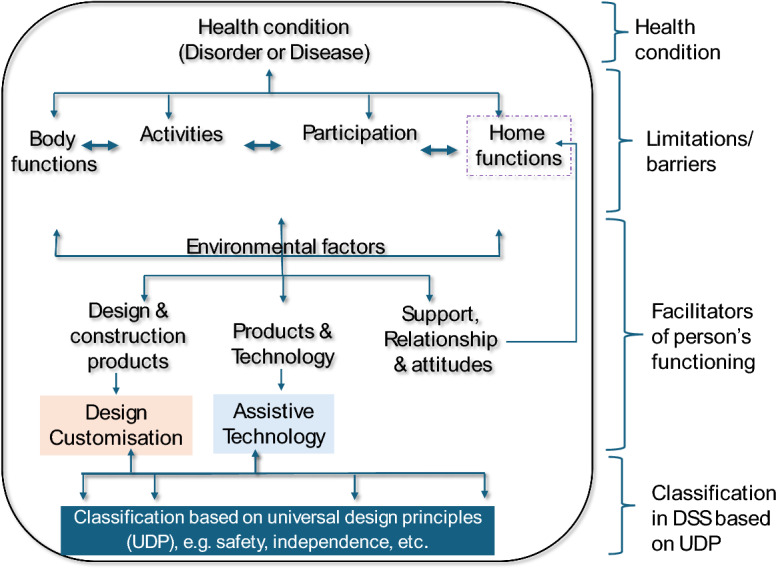
Project implementation framework. Source: Authors’ own work

Finally, the adaptation housing factors were added to the framework, focusing on key home features so that specific, not generic, recommendations can be made where applicable. Figure 1 presents the project implementation framework.

## Methodology

Development-oriented research is practical and relevant to real-world development challenges, seeking to provide evidence-based solutions [21]. In with that, this study relies on constructive research and a coproduction approach similar to earlier study of Oyegoke [32]. The five main steps of constructivism [23, 32] are used for concept design and the development of a construct implementation framework. The practical problem of delay in housing adaptation was identified as a practical and relevant problem in housing adaptations [33, 34]. A general and comprehensive understanding of the topic is carried out through a comprehensive review of the literature to define the knowledge gap and specify the research problem. The innovative construct is underpinned by theoretical justification and testing of solutions to demonstrate that the solution works and leads to a contribution to knowledge .

The constructivist approach uses an embedded coproduction and interpretive approach with high-value research decision-making distributed across the team. Coproduction shapes the research process in a less hierarchical and more distributed structure and provides the methodological flexibility with critical reflection in the research process to report how knowledge was produced. This research involved researchers, system developers, occupational therapists, practitioners and public sector workers to constitute valid knowledge-based in housing adaptations and recommendation of assistive technologies.

The research strategies relied upon are brainstorming and focus groups. Brainstorming is a creative technique and problem-solving approach normally used for idea generation. It is used in many idea-generation research studies [16]. The four steps of classical brainstorming as reported in Oyegoke et al. [32] is followed. Hennink (2014) states, “the purpose of a focus group is to gather perspectives” The focus group approach is also used to gather diverse experts’ perspectives and opinions about the ideas, evaluate and validate the proposed implementation framework. Stakeholder engagement was also used at the inception and testing phase of the development. The study is designed in conformity with all ethical issues around anonymity, confidentiality and informed consent. Brainstorming is used by the project team to generate ideas and draw links between them to find potential solutions. A two-level brainstorming was carried out; fortnightly at the project level and weekly by the research team during the six months of the feasibility project. This is complemented by 2 focus group exercises with external partners for their feedback.

### Datasets for the study

The dataset comprises structured records of various diseases and symptoms [44] that influence the recommendation of assistive technologies [22] and home adaptation solutions. The study relied on three distinct datasets with associated housing adaptations and assistive technologies. The first, Dataset-1, focused on individuals with Muscular Dystrophy (MD). This dataset formed the foundation of the study, providing insights into the health conditions and specific housing adaptation needs of individuals with MD. The second dataset, Dataset-2, expanded the scope to include data from individuals with five-additional diseases based on the most prevalent health conditions that lead to housing adaptation in the UK [30]. It includes Parkinson’s disease, Arthritis, Stroke-related disabilities, Motor neuron disease, and Multiple Sclerosis. This expansion enabled a deeper understanding of the diverse housing and assistive technology requirements for individuals with these conditions. The third dataset, Dataset-3, was the most comprehensive, incorporating data for over 500 different diseases, sourced from prominent databases like the NHS [29] and Mayo Clinic [27].

The mappings between diseases, symptoms, assistive technologies, and housing adaptations were constructed based on publicly available clinical and design guidelines and are intended for methodological feasibility evaluation rather than expert-labelled clinical ground truth. These mappings follow internationally recognised frameworks and standards, including the WHO International Classification of Functioning [44], ISO 9999:2022 (International Organisation for Standardisation 2022), and the Muscular Dystrophy UK Adaptations Manual [28]. The Building Regulations in Approved Document M on access to and use of buildings are also used in developing the database based on M4(1) visitable dwellings, M4(2) accessible and adaptable dwellings and M4(3) wheelchair user dwellings [40]. Data was also extracted from the Inclusive Housing Design Guide (Habinteg Housing Association, 2024), which advocates for designs that are usable by all people, regardless of ability. ML models, NLP techniques, and statistical analysis are integrated to develop an intelligent recommendation system for assistive technologies and housing adaptations.

In the datasets, each row represents unique data for a specific disease, including affected body functions such as cognitive functions, hearing, muscle power, and gait pattern. It also includes limitations in activities such as vision impairment, difficulty walking, and limited mobility, along with environmental conditions within the home, such as accessibility, the presence of stairs, or the absence of an elevator. Based on this multidimensional input, the dataset maps suitable assistive technologies, including stair lifts, hearing aids, cognitive assistance devices, and voice-activated systems. This vast dataset allowed for an extensive analysis of various disabilities and medical conditions, improving predictions and classifications for personalised housing adaptations.

For the needs assessment module, datasets include:

- Detailed information on physical, cognitive, and sensory impairments.
- Specific mobility-related challenges (e.g., wheelchair dependency, difficulty navigating stairs).
- Recommended assistive devices and adaptations based on clinical guidelines. Datasets are sourced from reputable websites such as the NHS and Mayo Clinic and are periodically updated to ensure accuracy. The inclusion of these datasets allows the system to address a broad spectrum of user requirements effectively.
- The system also employs advanced NLP techniques, synonym mapping, and scalable database solutions to ensure accurate, consistent, and dynamic recommendations based on evolving data sources.

## System design, development, application and usability

**Fig. 2:**
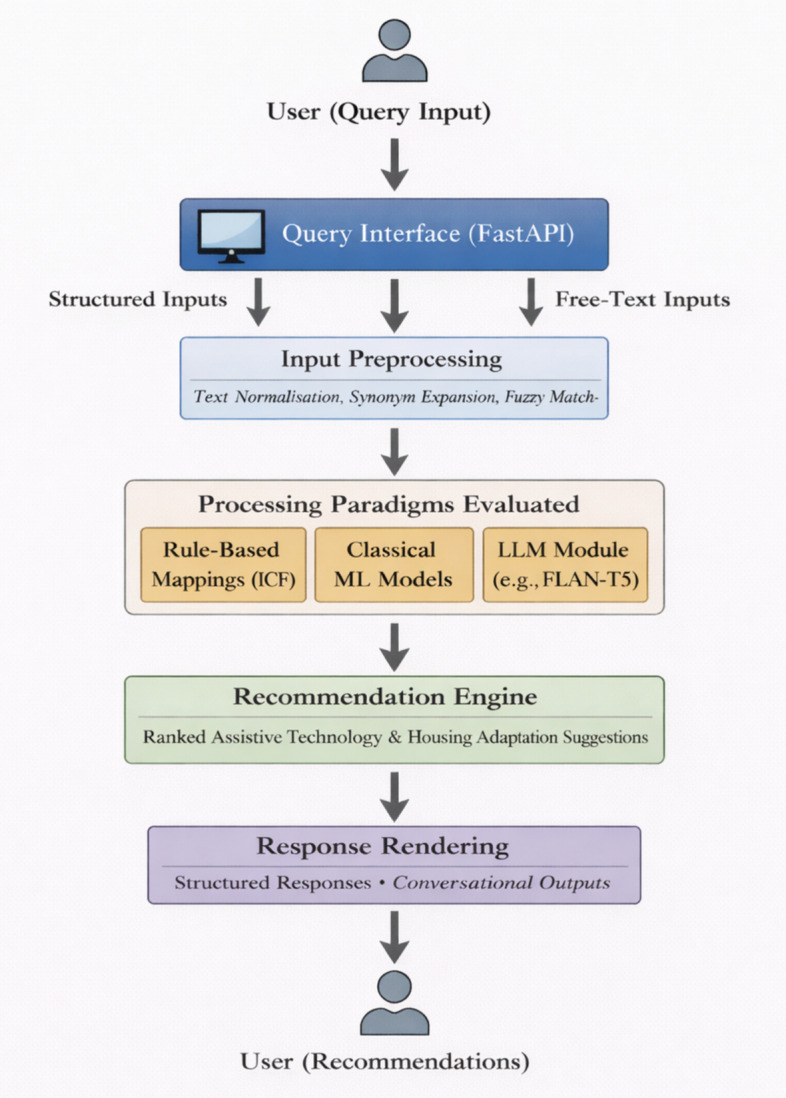
Overall architecture of the HomeAI-Enable feasibility framework

The platform is designed with a modular structure to ensure scalability, maintainability, and seamless functionality integration. Its core components include a user-friendly front-end interface built with HTML, CSS, and JavaScript for dynamic content updates, a backend powered by Python and FastAPI for processing user queries, and an NLP pipeline leveraging libraries like transformers and nltk for keyword extraction and fuzzy matching. The data management system processes datasets on diseases, symptoms, assistive technologies, and home adaptations, with the potential for integration with external APIs like the NHS or Mayo Clinic to ensure accurate, validated and updated medical information. The information structure follows a relational model, linking diseases to symptoms, assistive technologies, and home adaptations to provide personalised recommendations. The workflow begins with user query submission, followed by NLP-based preprocessing and data mapping. It concludes with the retrieval of relevant information and the dynamic presentation of results on the front end. This design supports a secure, efficient, and user-centric platform with the ability to scale and adapt as requirements evolve. Figure 2 illustrates the layered architecture adopted in this feasibility study to enable comparative evaluation of rule-based, classical machine learning, and LLM-driven recommendation paradigms within a unified decision support pipeline. The architecture follows a layered, modular pipeline comprising (i) user interaction, (ii) NLP preprocessing, (iii) dataset mapping and retrieval, (iv) multi-paradigm inference (rule-based, classical ML, and LLM-based modules), and (v) response rendering. Data flow proceeds from the user query to preprocessing (normalisation, keyword extraction, synonym expansion, fuzzy matching), then to paradigm-specific processing over the curated datasets, after which recommendations are generated and returned via the web interface.

## Implementation and results of the feasibility study

The technical feasibility of developing the database and system involves different technical approaches to evaluate the prospective implementation and development of the project. It examines the applicability of NLP and fuzzy logic for causation and correlation on all the three datasets. Thereafter, ML models were applied for Dataset-2, and advanced ML models leveraging Large Language Model (LLM) like FLAN-T5 and fuzzy matching techniques for Dataset-3. ML model was not applied to Dataset-1 because it exclusively focuses on Muscular Dystrophy and contains a limited, manually curated set of disease-related activities and assistive technologies. The dataset is small in scope, structurally simple, and intended for direct lookup and educational mapping, not prediction. As such, traditional rule-based keyword matching and domain logic are sufficient to retrieve relevant recommendations.

### Results from dataset-1

Table 1 presents a sample data line from Dataset-1, illustrating the effects of Muscular Dystrophy on individuals’ body functions, daily activities, and the corresponding environmental and technological interventions required. The Dataset-1 (see Appendix 1) highlights how impairments such as emotional regulation, balance, and mobility impact tasks like walking, communication, and self-care. It links these challenges to specific housing adaptations such as ramps, wide doorways, tactile flooring, and adaptive bathroom features, and assistive technologies including mobility aids, communication devices, and posture support tools.

**Table 1:** Dataset 1 for muscular dystrophy disease

Specific disease	Body functions	Activities and participation	Home function with ICF code	Assistive technology with ICF code
Muscular Dystrophy	Temperament and personality functions (b126)	Difficulty managing emotional responses and stress due to physical limitations. (d230)	Environmental controls and safe moving/handling solutions. (125)	Psychological support and therapy devices. (e125)
Muscular Dystrophy	Emotional functions (b152)	Limited participation in social activities due to frustration and mood swings. (d240)	Wide doorways and ramps to improve access for social spaces, controlled heating. (135)	Social participation tools such as adaptive communication devices. (e125)
Muscular Dystrophy	Perceptual functions (b156)	Impaired perception can affect day-to-day interactions and mobility. (d250)	Tactile floor coverings, motion-sensor lighting, defined pathways. (150)	Mobility aids for safe interaction (canes, wheelchairs). (e135)
Muscular Dystrophy	Mental function of sequencing complex movements (b176)	Difficulty with tasks requiring coordination and complex movements. (d435)	Adaptive kitchen tools, grab bars in bathrooms, and touch-sensitive controls. (155)	Assistive tools for fine motor skills in cooking and other tasks. (e135)
Muscular Dystrophy	Seeing functions (b210)	Challenges in interacting with the environment due to visual impairments. (d110)	Vertical through-floor lifts and enhanced lighting to aid navigation. (120)	Visual aids and navigation tools. (e120)
Muscular Dystrophy	Hearing functions (b230)	Limited mobility and communication due to hearing difficulties. (d330)	Flashing light systems for alarms, and voice-activated environmental controls. (110)	Hearing aids and visual communication systems. (e125)
Muscular Dystrophy	Vestibular functions (b235)	Balance issues causing falls and difficulty with standing or walking. (d455)	Portable ramps, grab bars, non-slip floors, stairlifts. (150)	Fall prevention devices like walkers and grab bars. (e150)
Muscular Dystrophy	Proprioceptive function (b260)	Reduced ability to maintain body posture and stability during movement. (d450)	Electric profiling beds and adjustable seating for posture support. (135)	Posture-support devices like chairs and beds. (e135)

Using the limited data presented in Dataset-1, a basic prediction model was developed to explore the relationship between impairments caused by Muscular Dystrophy and appropriate housing adaptations or assistive technologies. This method undergoes several key preprocessing steps to ensure the dataset is usable for predictions. First, fuzzy matching techniques are employed to match user inputs, such as disease symptoms or activity limitations, to the closest entries in the dataset. A simple decision tree classifier was used to associate activity limitations with possible support measures. However, due to the very small dataset and the focus on a single condition, the model performed poorly, with prediction accuracy falling below 20%. The output shown below reflects the limitations of the dataset and reinforces the need for broader, multi-condition data to support effective and reliable prediction.

Based on the first row of Dataset-1, which included temperament and personality issues along with challenges in emotional regulation and stress management, the model was tested to evaluate its predictive capability. It recommended generic interventions such as calming lighting, quiet zones, voice-activated controls, and therapy support tools. However, the actual expert-provided recommendations were more specific and clinically grounded, including environmental controls for safe handling and targeted psychological support devices. With only one accurate match between model output and expert suggestions, the resulting accuracy was under 20%. This outcome highlighted the model’s limited ability to account for complex emotional and behavioural profiles when trained on a narrowly scoped dataset. As shown in Fig. 2, the system identifies the most relevant input keyword and generates tailored assistive technology and housing recommendations, yet the gap between surface-level semantic matches and clinically nuanced needs remains a key limitation.

**Fig. 3:**
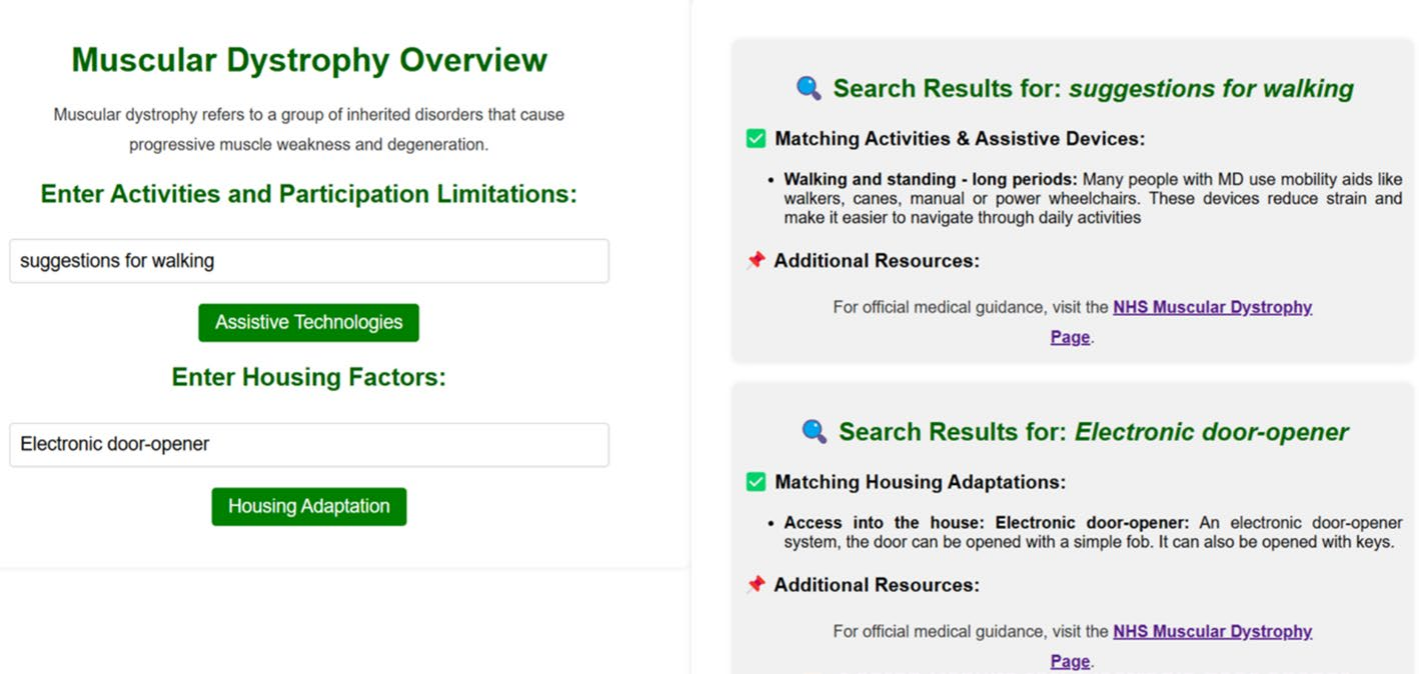
Personalised suggestions for muscular dystrophy for assistive technologies

Based on the first row of Dataset-1, the model was tested using temperament and personality issues alongside difficulties in managing emotional responses and stress as input. It predicted basic interventions such as calming lighting, quiet zones, voice activated controls, and therapy support tools. However, the actual expert recommendations included more specific solutions like environmental controls for safe handling and psychological support devices. With only one match, the result once again demonstrated less than 20% accuracy, highlighting the model’s limited capacity to reflect complex emotional and behavioural needs with such a narrow dataset. The system generates recommendations based on the relationships between the datasets. Figure 3 highlights the most relevant keyword from the input and offers tailored recommendation for assistive technologies and home adaptations suited to the individual’s needs, ensuring that they receive relevant, practical advice to improve their quality of life.

### Results from dataset-2

Instead of focusing on one disease at a time, this dataset- 2 explores five conditions: Arthritis, Multiple Sclerosis, Parkinson’s disease, Muscular Dystrophy, and Stroke (See Table 2 for sample data line from Dataset-2). Five different ML models were applied to Dataset-2 (see Appendix 2) to predict assistive technology and housing adaptation: a SMOTE-XGBoost, dual Random Forest classifiers, parallel XGBoost classifiers, a Stacked Ensemble model, and a Feature Optimised Voting model. These models were selected to cover a range of predictive strategies suited for structured, categorical, and imbalanced data. XGBoost and Random Forest were chosen for their strong performance in handling nonlinear relationships and high-dimensional inputs [6, 11]. Ensemble approaches such as stacking and voting leverage model diversity to improve robustness and generalisation [24]. Logistic Regression was included within ensemble layers due to its interpretability, and Synthetic Minority Over-sampling Technique (SMOTE) was consistently applied to mitigate class imbalance across all experiments [10]. Together, these models allow for a comparative evaluation between joint and independent target prediction strategies and between single and ensemble model architectures.

**Table 2:** Dataset 2 for five specific diseases

Sl No	ID	Specific disease	Body functions	Activities and participation	Home functions	Assistive technology	Customised housing design
1	2	Arthritis	Cognitive functions (b164)	Vision impairment (d110)	Multi-level home (e150)	Cognitive Assistance Device (e125)	Smart Home Integration (e155)
2	4	Arthritis	Hearing functions (b230)	Vision impairment (d110)	Accessible (e150)	Hearing Aid (e125)	Accessible bathroom (e155)
3	258	Motor Neurone Disease	Vision functions (b210)	Vision impairment (d110)	Accessible (e150)	Wheelchair Ramp (e120)	Stairlift installation (e155)
4	271	Motor Neurone Disease	Hearing functions (b230)	Cognitive decline (d163)	Stairs present (e150)	Hearing Aid (e125)	Ramp installation (e155)
5	83	Multiple Sclerosis	Cognitive functions (b164)	Limited mobility (d455)	Accessible (e150)	Cognitive Assistance Device (e125)	Stairlift installation (e155)
6	85	Multiple Sclerosis	Vision functions (b210)	Cognitive decline (d163)	Accessible (e150)	Wheelchair Ramp (e120)	Stairlift installation (e155)
7	469	Stroke-related disabilities	Vision functions (b210)	Vision impairment (d110)	No elevator (e150)	Cognitive Assistance Device (e125)	Stairlift installation (e155)
8	472	Stroke-related disabilities	Muscle power functions (b730)	Limited mobility (d455)	Old building (e150)	Hearing Aid (e125)	Enhanced lighting (e155)
9	520	Parkinsons Disease	Hearing functions (b230)	Limited mobility (d455)	Accessible (e150)	Cognitive Assistance Device (e125)	Smart Home Integration (e155)
10	526	Parkinsons Disease	Muscle power functions (b730)	Vision impairment (d110)	Accessible (e150)	Wheelchair Ramp (e120)	Stairlift installation (e155)

#### SMOTE-XGBoost (joint label) method

This method aims to predict one combined outcome by merging both assistive technology and customised housing design into a single label. Categorical input features like specific disease, body functions, activities and participation, and home functions are converted using one-hot encoding. To deal with class imbalance in the combined target column, SMOTE is used. The dataset is then split into training and testing sets, and an XGBoost classifier is trained with hyperparameter tuning through RandomizedSearchCV. This approach works well for joint prediction, keeps the relationship between the two targets intact, and simplifies the process. On the downside, it is not very flexible, as highlighted in the study by [46], where joint label modelling was found to limit flexibility and increase the risk of compound prediction errors. The output is a single label that includes both the assistive technology and housing recommendation, which can sometimes be confusing, such as “Hearing Aid plus No elevator.” The model output reached an accuracy of 12.80%.

#### Dual independent random forest modelling method

This approach treats assistive technology and customised housing design as two separate targets. After applying one-hot encoding and scaling the features using StandardScaler, SMOTE is applied individually to each target to balance the dataset. Two Random Forest classifiers are trained, and RandomizedSearchCV is used to fine-tune the hyperparameters. This method provides flexibility and clarity by amodelling each target on its own, and it handles class imbalance well. However, it demands higher computational resources and does not ensure alignment between the two outputs, a limitation also noted by Borchani et al. [4]. The output gives two unconnected predictions, one for assistive technology and another for housing design. For example, it predicted “Voice-activated devices” for assistive technology and “Smart Home Integration” for housing. The accuracy is 23.53% for assistive technology and 23.04% for housing design.

#### Parallel XGBoost classification method

This method uses two separate XGBoost models to predict each target. The features are one-hot encoded, and the targets are label encoded. SMOTE is used for balancing, and StandardScaler ensures consistent scaling across the features. Hyperparameter tuning is done with RandomizedSearchCV. This approach delivers strong performance and better accuracy on the assistive technology than the previous two, thanks to XGBoost’s speed and optimisation. On the downside, it can be harder to interpret than simpler models, and tuning can be time-consuming. The method produces two independent predictions with an accuracy of 26.24% for assistive technology and 20.59% for housing design.

#### Stacked ensemble learning method

This technique combines several classifiers, including XGBoost, Random Forest, and Logistic Regression, using a Stacking Classifier. Interaction features are added to improve learning, and GridSearchCV is used to tune each layer of the model. The evaluation is done using a standard train-test split. This method benefits from ensemble learning, capturing complex patterns by combining the strengths of different models. However, it is more complex to build and takes longer to train and debug. The output includes two well-tuned predictions for assistive technology and housing design. The accuracy is 22.62% for assistive technology and 17.65% for housing design. While both predictions are well optimised independently, the model treats assistive technology and housing adaptation as separate targets. As such, the outputs are not jointly conditioned or constrained, which may lead to combinations that are not directly coordinated. For instance, the model might recommend “Voice activated devices” for assistive technology and “Smart Home Integration” for housing design. This is in contrast to joint label approaches, where a combined prediction is generated and the interdependence between the two recommendations is preserved. The stacking method offers flexibility but does not inherently ensure alignment between outcomes.

#### Feature optimised voting classifier method

This method uses Recursive Feature Elimination to select the top 20 features. A Voting Classifier combining XGBoost and Random Forest is applied using soft voting. Separate pipelines are created for each target, and GridSearchCV tunes the models. This approach improves interpretability and balances performance, though it does not always outperform standalone models. The accuracy achieved is 25.79% for assistive technology and 19.61% for housing design.

In Dataset-2, five (5) ML models were employed to predict assistive technology and housing adaptation solutions, including a SMOTE-XGBoost, dual Random Forest classifiers, parallel XGBoost classifiers, a Stacked Ensemble model, and a Feature Optimised Voting model. These models were selected to explore different strategies for handling structured, categorical and imbalanced data, with ensemble methods offering improved robustness through model diversity. While these approaches performed reasonably well, demonstrating accuracies up to 26.24%, they were fundamentally limited by their reliance on disease based static labels, which constrain the adaptability and personalisation of predictions. These accuracy levels are not intended to represent clinically or policy-usable performance but are interpreted here as diagnostic evidence of the structural limitations of disease-labelled supervised learning in this application domain. To address these limitations and enhance the relevance of outputs, the study transitions to a more advanced model on a comprehensive dataset (Dataset-3) which adopts a more flexible, symptom-driven approach.

### Results from dataset-3

The subset from Dataset-3, as shown in Table 3, contains structured records of individuals with various health conditions, focusing on personalised accessibility needs, and is compiled from publicly accessible databases such as the NHS and the Mayo Clinic. It consists of three real-time datasets, each with over 500 entries. The first dataset (Dataset-3a; see Appendix 3) maps diseases to their associated symptoms. The second (Dataset-3b; see Appendix 4) and third (Dataset-3c; see Appendix 5) link symptoms to assistive technologies and home adaptations, respectively. These datasets serve dual purposes: the first supports accurate diagnosis through symptom understanding, while the latter two provide tailored intervention recommendations.

**Table 3:** Dataset 3 for multiple diseases

Disease	Symptoms	Symptoms	Assistive technology	Symptoms	Home functions
Abdominal aortic aneurysm	Tummy pain, Back pain, a Pulsing feeling in tummy	Abdominal Cramps	Electric heating pads (e.g., Sunbeam), TENS devices (e.g., Omron)	Abdominal cramps	Comfortable seating with heat pads nearby
Acanthosis nigricans	Patches of skin that are darker and thicker than usual	Abdominal Pain	Electric heating pads (e.g., ThermaCare)	Abdominal pain	Adjustable chairs with lumbar support
Achalasia	Difficult to swallow food	Abnormal Vaginal Bleeding	Absorbent pads (e.g., Always), menstrual cups (e.g., DivaCup)	Abnormal vaginal bleeding	Easily washable beddings and surfaces
Acromegaly	Enlarged hands and feet, facial changes, joint pain, fatigue	Aching in Legs	Leg supports (e.g., Vive Leg Rest Pillow)	Aching in legs	Leg elevation furniture and easy-access seating
Acute cholecystitis	Upper right abdominal pain, nausea, vomiting, fever	Acid Reflux	Bed wedge pillows (e.g., MedCline)	Acid reflux	Inclined beds or bed risers to elevate the head
Acute kidney injury	Reduced urine output, fatigue, shortness of breath, confusion	Acne	Blue light therapy devices (e.g., Foreo Espada), SkinGuru AI Skin Scanner	Acne	Hypoallergenic pillowcases and bedding
Acute lymphoblastic leukaemia	Fatigue, pale skin, easy bruising, fever, bone pain	Anxiety in Open or Crowded Spaces	Noise-cancelling headphones (e.g., Bose QuietComfort), calming apps (e.g., Calm), weighted blankets (e.g., Gravity Blanket)	Anxiety in open or crowded spaces	Calming colours and sensory-friendly zones
Acute myeloid leukaemia	Fatigue, fever, easy bruising, weight loss	Back Pain	Posture correctors (e.g., Upright Go 2), wearable posture sensors (e.g., Lumo Lift)	Back pain	Ergonomic office setup with proper back support

The system captures correlations through structured mappings across diseases, body functions and interventions using ML pipelines, and supports causal reasoning by following a directional flow from symptom to solution. This is further enhanced by deep learning models such as DistilGPT2, FLAN-T5, which enable context-aware and personalised recommendations. On this foundation, eleven (11) intelligent retrieval and recommendation models such as TF-IDF and Cosine Similarity, Fuzzy Matching with Tabular Output, Flask based Web application with fuzzy matching, Real-Time Suggestions with JavaScript and Flask, Conversational Flask App with Unified Input, AI-Powered Query Understanding with Synonym Mapping and GPT-based Responses, Context-Aware Matching with Dynamic Conversational Templates, Flexible Unified Search with Disease/Symptom Differentiation, Enhanced Synonym Mapping and LLM-Driven Conversational Interface, Dynamic Response Generator with Enhanced Dataset Integration, AI-Powered Multitiered Query Resolution with Real-Time Deployment were developed, each supporting a different level of user input, from structured disease labels to natural language queries. The following models demonstrate increasing interactivity, adaptability and user-friendliness, ranging from vector-based semantic search to conversational large language model interfaces. Ultimately, the AI-powered multitiered query resolution model integrated with FLAN-T5 [35] was selected as the best-performing system due to its flexibility, robustness, and tailored natural language responsiveness, setting the stage for system development.

#### TF-IDF and cosine similarity method

**Fig. 4:**
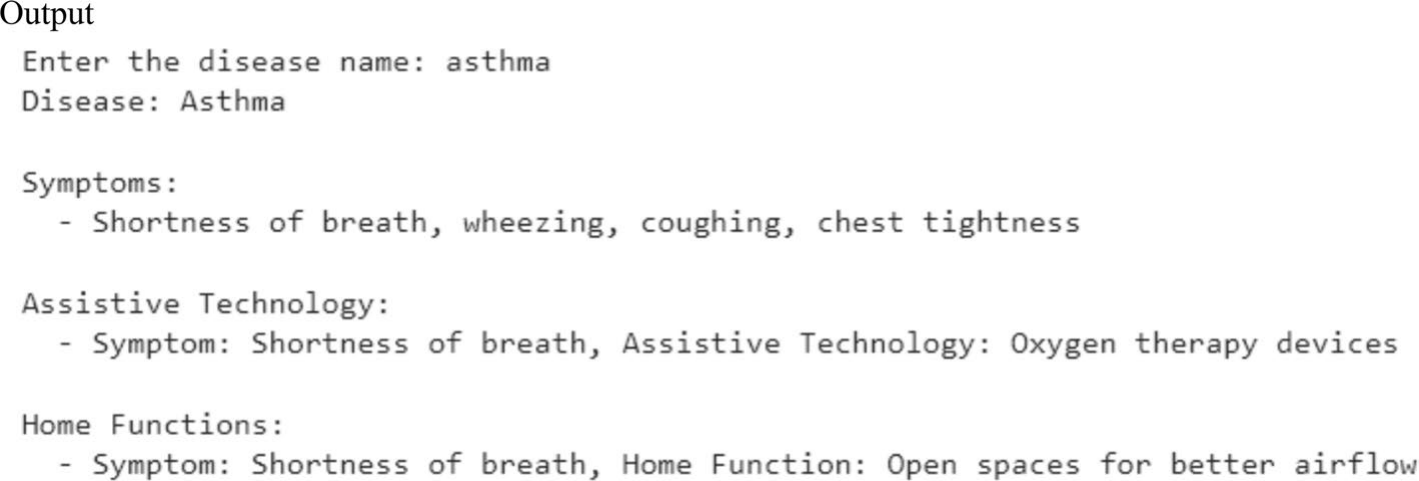
Personalised recommendations for asthma disease

This method extracts disease-specific recommendations using a semantic approach. The system reads from three structured Excel sheets: diseases linked to symptoms, symptoms mapped to assistive technologies, and symptoms mapped to home functions. After preprocessing the text (lowercasing and trimming), it uses TF-IDF to vectorise all symptom descriptions. When a disease is entered, its associated symptoms are retrieved, and cosine similarity is computed to find the closest matching symptoms in the assistive and home function datasets. If the similarity score is above a certain threshold (e.g., 0.5), the corresponding assistive technology or home adaptation is recommended. This approach captures semantic similarity even when the wording differs, making it effective for handling varied and unstructured textual input [25]. It performs well without requiring manual tagging, leveraging language understanding to identify relevant matches across symptoms, assistive technologies, and home adaptations. However, as the first method in the sequence, it relies heavily on clean and complete input text, in contrast to later models that are designed to handle unstructured or conversational input. When symptom descriptions are inconsistent or unclear, the system may produce weaker matches. The output, as shown in Fig. 4, consists of lists of relevant symptoms, assistive technologies, and home functions, all linked through computed similarity scores.

#### Fuzzy matching with tabular output method

**Fig. 5:**
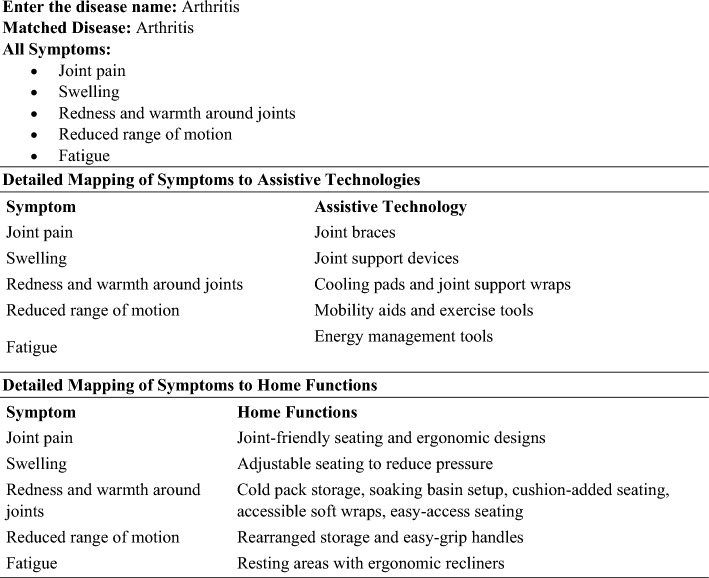
Detailed tabular mapping of arthritis based on fuzzy matching with tabular method

This method enhances usability by combining fuzzy string matching with tabular display. Using fuzzywuzzy, the system matches approximate strings between disease or symptom inputs and stored values. After loading and preprocessing the Excel sheets, it searches for the closest matching disease and retrieves related symptoms. Then, for each symptom, it finds matching assistive technologies and home functions using fuzzy logic. The results are displayed in neatly formatted tables using the tabulate library. This method is highly tolerant of typos and slight variations in user input, making it user-friendly and robust for imperfect text entries. The results are easy to read and interpret due to clear table formatting, which presents information in an organised way. However, the approach relies solely on string similarity for matching, which can sometimes lead to incorrect associations, especially when symptom names are very similar but refer to different conditions. The output as shown in Fig. 5, includes formatted tables that list matched symptoms along with their recommended assistive technologies and home adaptations.

#### Flask based web application with fuzzy matching method

**Fig. 6:**
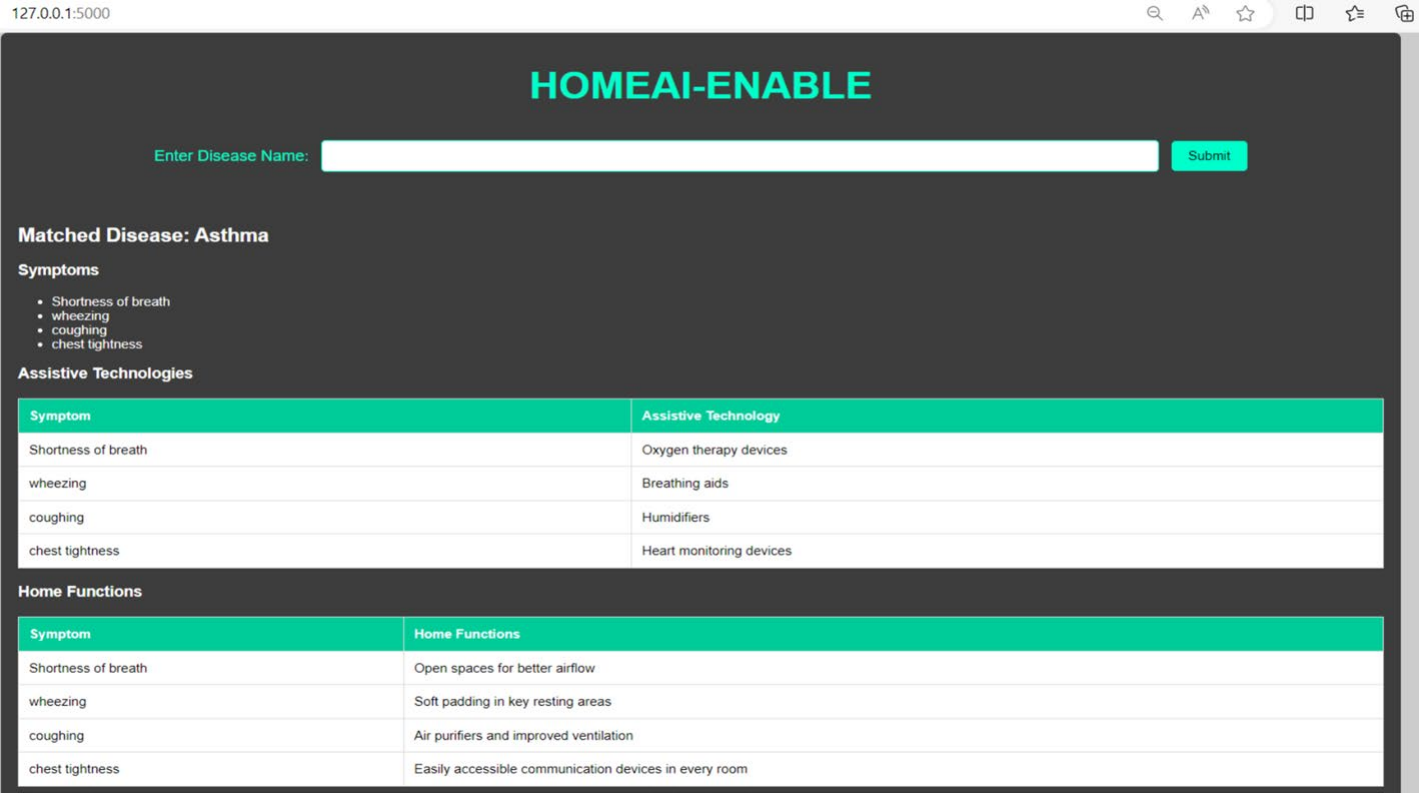
GUI overview of asthma disease with personalised assistive technologies and home functions

This method creates a user-friendly web app using Flask that allows real-time querying of disease-related assistive solutions. Users enter a disease name into a web form, and the backend applies fuzzy string matching to find the closest disease in the dataset. It then retrieves symptoms and maps each one to relevant assistive technologies and home functions. Results are presented on a visually styled HTML page for accessibility. This method is interactive and easy to use, offering a user-friendly interface that effectively handles fuzzy or imprecise input. It delivers clean, structured output, making it simple for users to understand the results. However, it requires server hosting and some additional setup, which may not be ideal for quick offline use. It is also limited to text input through a single disease search box, which can restrict more complex queries. The output, as shown in Fig. 6, is a web page displaying the matched disease, associated symptoms, relevant assistive technologies, and suggested home adaptations.

#### Real-time suggestions with JavaScript and flask

**Fig. 7:**
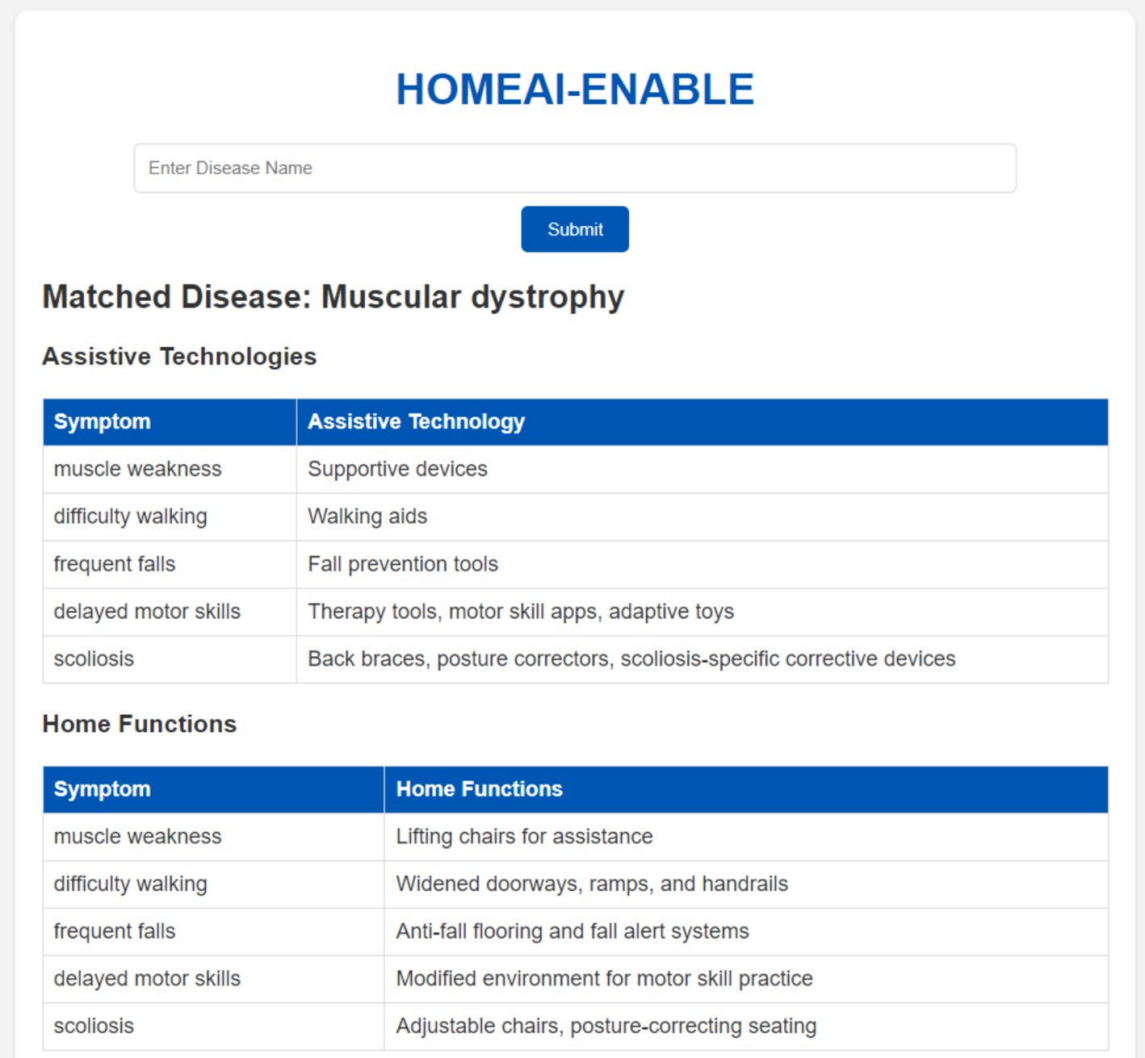
A more user-friendly GUI overview of muscular dystrophy recommendations

This method upgrades the user interface by integrating JavaScript for real-time disease name suggestions. As the user types, suggestions appear instantly using asynchronous calls to a Flask endpoint. When a suggestion is selected, it populates the form and triggers disease processing like in Method 3. The backend uses fuzzy logic to match symptoms and return detailed results. This feature enhances the user experience by minimising typing errors and reducing the effort required to search, thanks to its dynamic autocomplete functionality. It is fast and responsive, allowing users to quickly access disease-specific assistive solutions. However, it requires proper coordination between the frontend and backend components, making the implementation slightly more complex than static interfaces. The output, as shown in Fig. 7, is an interactive search interface that offers real-time suggestions and rapid access to relevant assistive technologies and home adaptation recommendations.

#### Conversational flask app with unified input

**Fig. 8:**
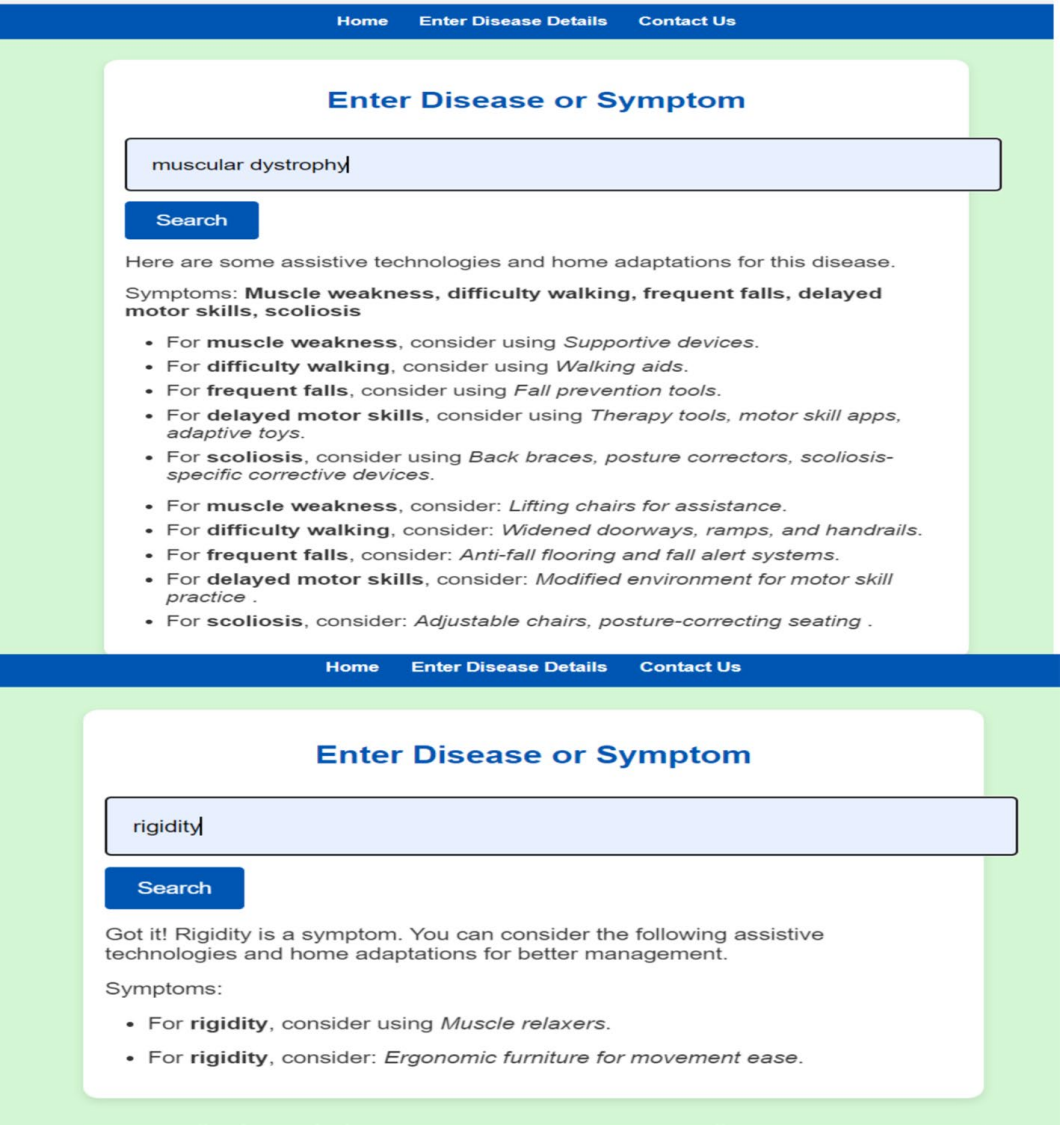
Overview of muscular dystrophy, along with a separate symptom search functionality

This method builds on earlier designs by allowing the user to enter either a disease or a symptom into a single search box. The system determines whether the input is a disease or symptom using fuzzy matching, then returns related assistive and home recommendations. The interface mimics a conversational style, offering bullet-point suggestions in a natural tone. Text preprocessing ensures consistent matching across all datasets. This approach is extremely flexible and user-friendly, allowing users to input either diseases or symptoms while still receiving meaningful results. It offers an intuitive and engaging experience, making the output feel more personalised and relevant. However, it requires additional logic to accurately distinguish between different types of input, and there can be potential ambiguity if the input is too broad or generic. The output, as shown in Fig. 8, is a personalised response that lists suitable assistive technologies and home adaptations based on the user’s entry, whether it is a specific disease or a general symptom.

#### AI-powered query understanding with synonym mapping and GPT-based responses

This method uses a keyword extraction and synonym-matching approach to interpret user queries such as “Suggestions for Parkinson’s disease” or “Help with muscle weakness.” The input undergoes advanced preprocessing, including lowercasing, regex cleanup, compound word splitting, and stopword removal, and is matched against a precompiled list of diseases and symptoms using fuzzy logic and NLTK’s WordNet synonyms. Once a relevant match is found, the system fetches associated assistive technologies and housing adaptations from two datasets. Additionally, Hugging Face’s DistilGPT2 model is used to generate a friendly, conversational explanation to guide the user. The application is built using FastAPI and is deployed via ngrok, making it publicly accessible through a styled, interactive web interface.

**Fig. 9:**
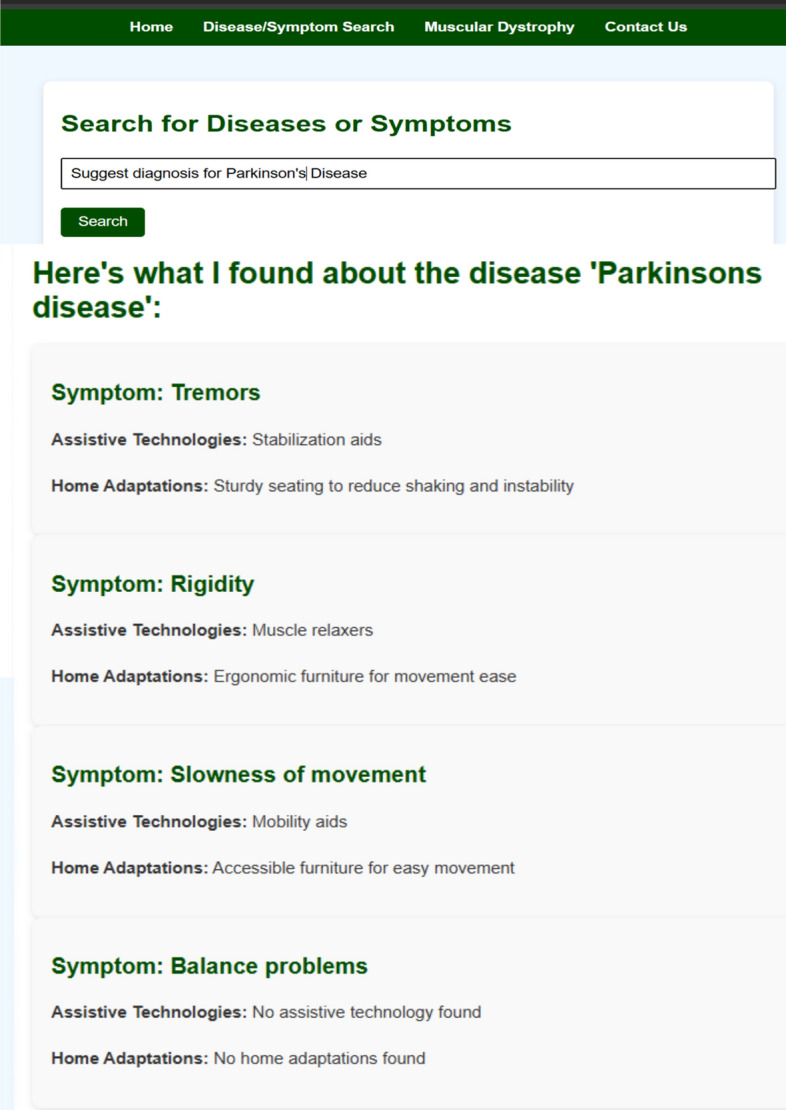
Interactive input query-based personalised recommendations for Parkinson’s disease symptoms

This method effectively handles vague or conversational input, allowing users to express their concerns in natural language. It integrates AI-generated text to provide more human-like explanations, making the interaction feel supportive and approachable. The inclusion of robust synonym detection enhances matching accuracy, even when exact terms are not used. However, responses may vary slightly between requests due to the nature of language generation, and processing can be slower because of the underlying NLP and model loading requirements. The output, as shown in the Fig. 9, is a friendly, AI-generated explanation that highlights the matched keyword and provides related assistive technologies and home adaptation suggestions.

#### Context-aware matching with dynamic conversational templates

**Fig. 10:**
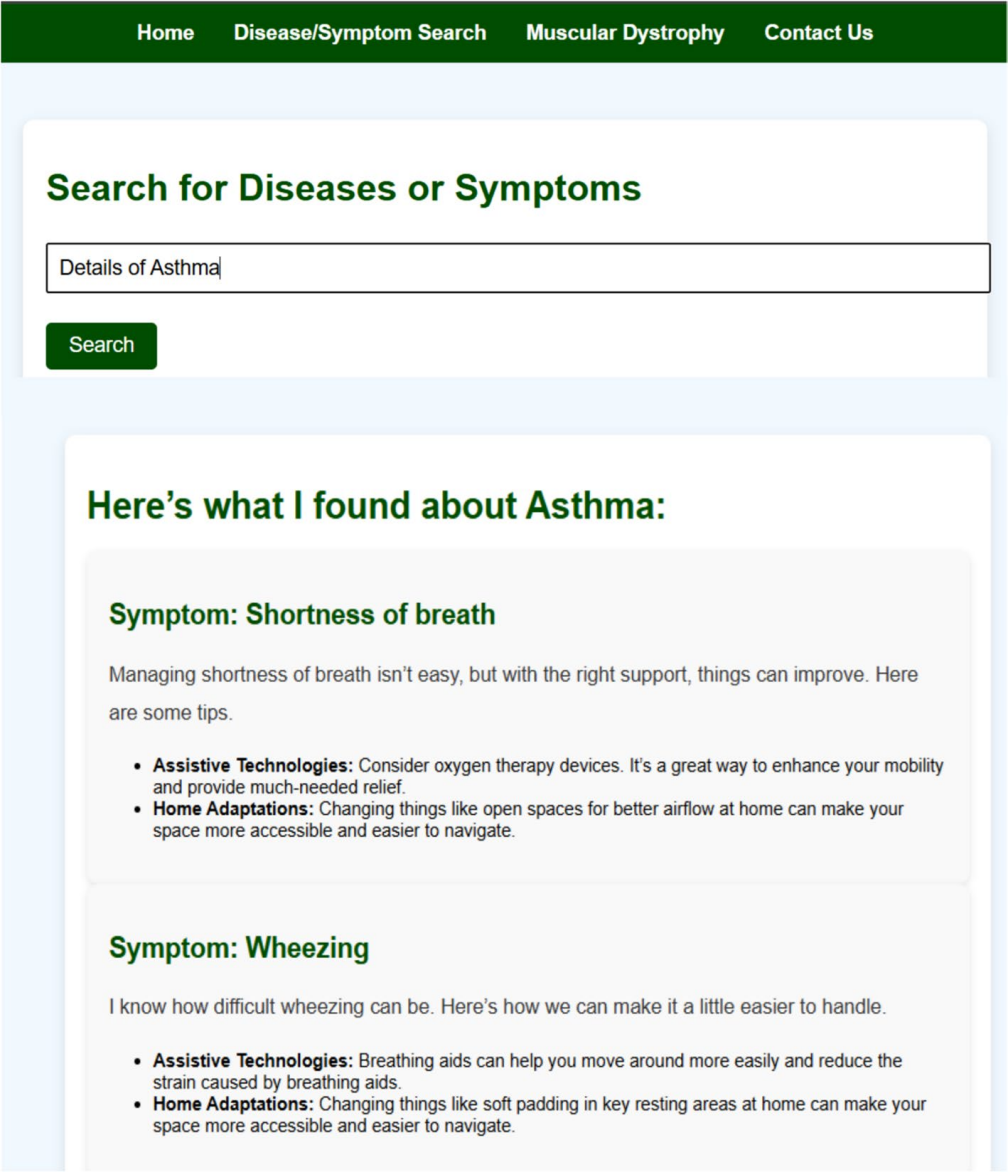
Conversational personalised recommendations for asthma disease

This method improves upon Method 6 by adding dynamic response generation using rotating conversational templates. It still uses synonym mapping, fuzzy matching, and keyword extraction, but now applies contextual token analysis to improve the accuracy of input interpretation even for misspelt or compressed terms like “Parkinsonsdisease”. Once a match is found, the system retrieves symptoms and suggests assistive technologies and home adaptations accordingly. Instead of static output, the method uses randomised templates for both assistive and home recommendations, making responses feel more natural and empathetic. Like Method 6, it is deployed with FastAPI and ngrok and offers a clean HTML-based UI. This method is highly user-centric and emotionally engaging, offering a more relatable experience by interpreting complex or non-standard input with greater accuracy. It supports diverse and natural response flows, making interactions feel more human and tailored. However, the use of randomisation and dynamic phrasing can reduce consistency across sessions, and additional logic is needed for effective context analysis and response generation. The output, as shown in the Fig. 10, is a personalised response that identifies a matched disease or symptom, followed by natural-language suggestions for relevant assistive technologies and home modifications.

#### Flexible unified search with disease/symptom differentiation

**Fig. 11:**
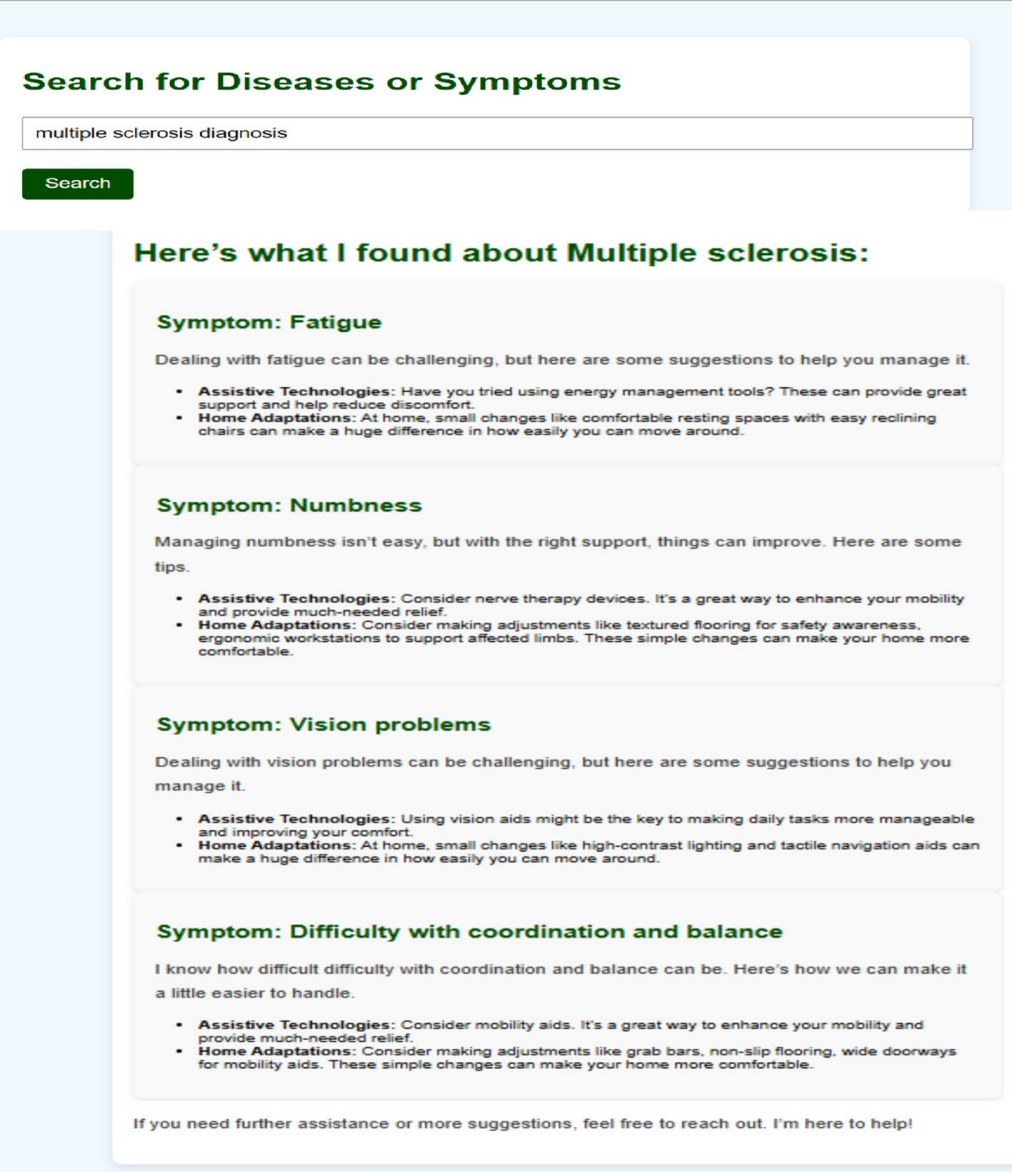
LLM-driven personalised recommendations for multiple sclerosis disease and symptoms in a single search box

This method builds on earlier designs by allowing the user to enter either a disease or a symptom into a single search box. The system determines whether the input is a disease or symptom using fuzzy matching, then returns related assistive and home recommendations. The interface mimics a conversational style, offering bullet-point suggestions in a natural tone. Text preprocessing ensures consistent matching across all datasets. This method is extremely flexible and user-friendly, capable of processing both disease names and symptom descriptions. It allows users to interact in a way that feels intuitive and natural, with outputs that are clear, engaging, and personalised. However, it requires additional logic to correctly interpret and distinguish between input types, and there’s a risk of ambiguity if the input is too broad or generic. The output, as shown in the Fig. 11, consists of a tailored response that lists appropriate assistive technologies and home adaptations based on the user’s input, whether it originates from a disease or a symptom.

#### Enhanced synonym mapping and LLM-driven conversational interface

This method introduces a fully integrated FastAPI-based web application that dynamically processes user queries to suggest personalised assistive technologies and home adaptations. It begins by applying advanced preprocessing techniques, including stopword removal, regex normalisation, and compound word splitting, to clean and standardise the user input. Keyword extraction is powered by context-aware scoring and fuzzy matching, using both direct matches and synonym lookups via NLTK’s WordNet. If a symptom or disease is identified, the system fetches relevant assistive devices and housing suggestions from an enriched dataset. What sets this method apart is its integration with Hugging Face’s DistilGPT2 model for real-time, empathetic response generation. The system utilises prebuilt and randomised conversational templates, ensuring responses feel fresh, human-like, and engaging. It also features a clean, interactive UI with robust route handling and ngrok deployment for accessibility.

**Fig. 12:**
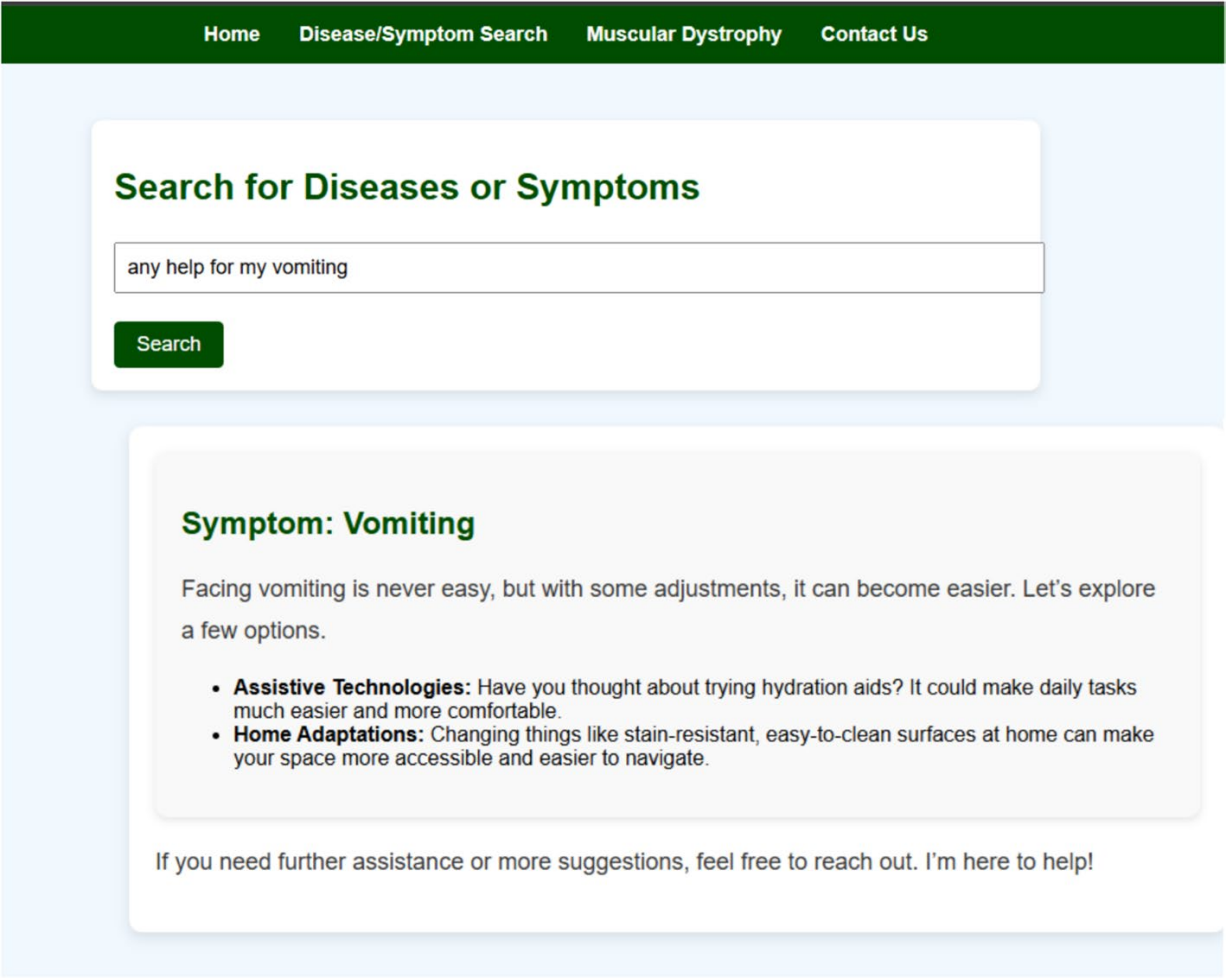
Empathetic personalised recommendations for vomiting symptoms

This method combines NLP, fuzzy logic, and synonym expansion to robustly handle a wide range of user input. It uses GPT-based text generation to produce empathetic and varied suggestions, enhancing the user experience with human-like interactions. The system is built for scalability and modular deployment via FastAPI, making it efficient for web-based applications. It handles both diseases and symptoms with flexibility, using context-sensitive keyword mapping to ensure relevant matches. However, the use of LLMs can lead to slight inconsistencies in responses, and the integration of multiple models and datasets increases processing time and memory demands. Additionally, fuzzy matching may reduce accuracy when the input is too vague or involves overlapping concepts. The output, as shown in the Fig. 12, is a dynamic, conversational HTML response tailored to the user’s query, featuring symptom-specific opening text, assistive technology suggestions, and home adaptation tips, all generated in natural, user-friendly language.

#### Dynamic response generator with enhanced dataset integration

**Fig. 13:**
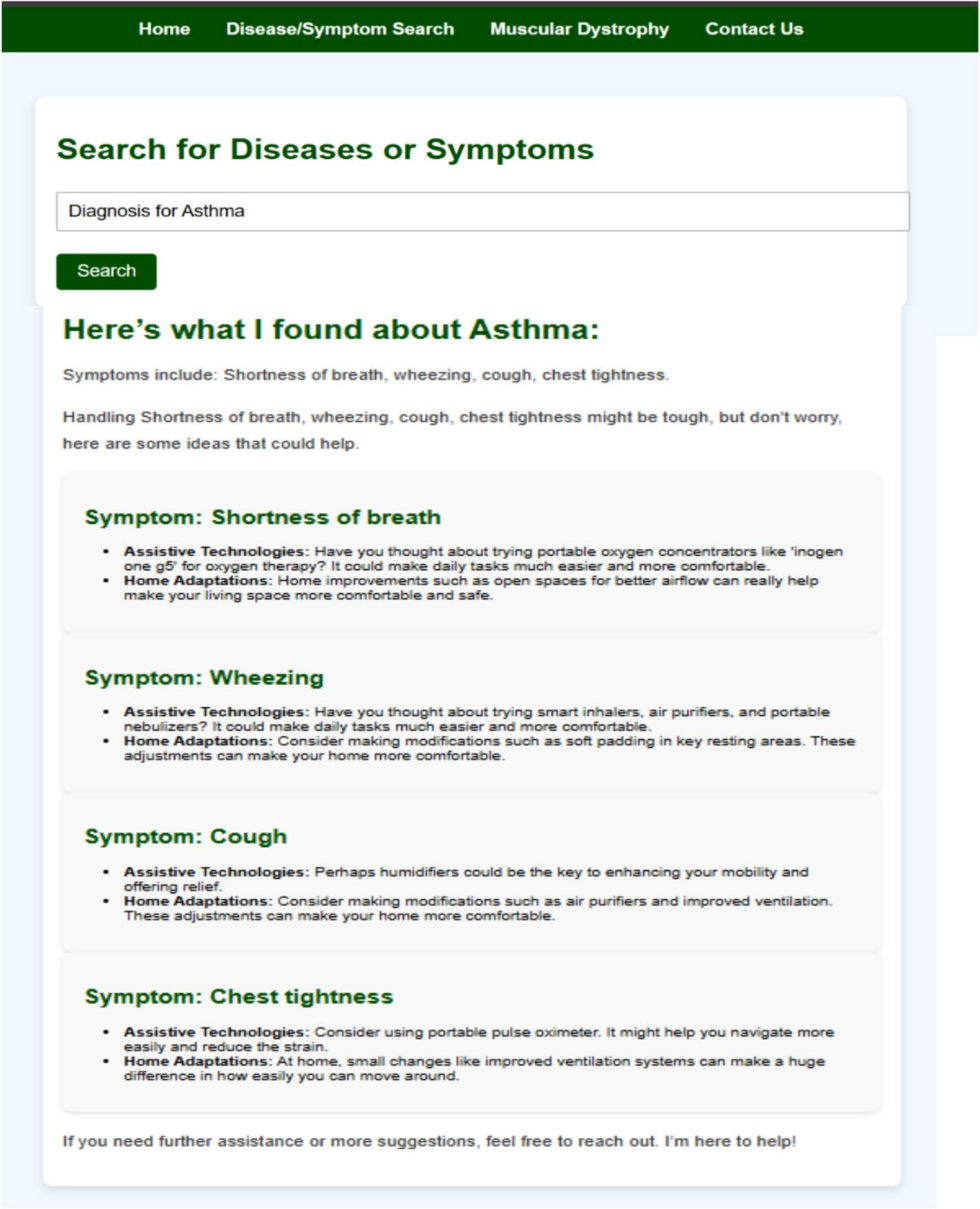
LLM (FLAN-T5) based personalised recommendations for asthma symptoms

This method introduces a major upgrade through the use of LLMs like FLAN-T5 model for generating conversational and context-aware suggestions. It combines multiple datasets (disease-symptom mappings, assistive technologies, and home adaptations), then uses advanced keyword extraction, including compound word breaking, synonym matching (via WordNet), and contextual token analysis to interpret complex user inputs. A FastAPI web interface allows real-time interaction, while fallback mechanisms ensure response variety using prewritten templates when the language model is unavailable. This method delivers smart, dynamic, and personalised responses, effectively handling complex, vague, or imprecise queries. By integrating broad and diverse datasets, it enhances coverage and improves the chances of generating relevant suggestions. The system excels in producing real-time, conversational responses that feel natural and engaging. However, the use of LLMs makes it more computationally intensive, and in some cases, the generated response may miss the user’s intent, especially if the input is highly ambiguous. The output, as shown in the Fig. 13, consists of a conversational reply with varied sentence structures, offering assistive technology and home adaptation suggestions related to the identified disease or symptom.

#### AI-powered multitiered query resolution with real-time deployment

This method integrates a multilayered NLP-driven query resolution architecture into a FastAPI web framework, designed to respond dynamically to user inputs across multiple tabs, such as Disease Search, Muscular Dystrophy, and Assistive Technology. The system leverages advanced keyword extraction, synonym expansion, fuzzy logic matching, and word-order-independent n-gram analysis to interpret queries even when phrased ambiguously. The FLAN-T5, LLM developed by Google research is used to generate empathetic, conversational responses, replacing static outputs. Pre-processed datasets from Excel sheets serve as the knowledge base for mapping diseases to symptoms, assistive tech, and housing adaptations.

**Fig. 14:**
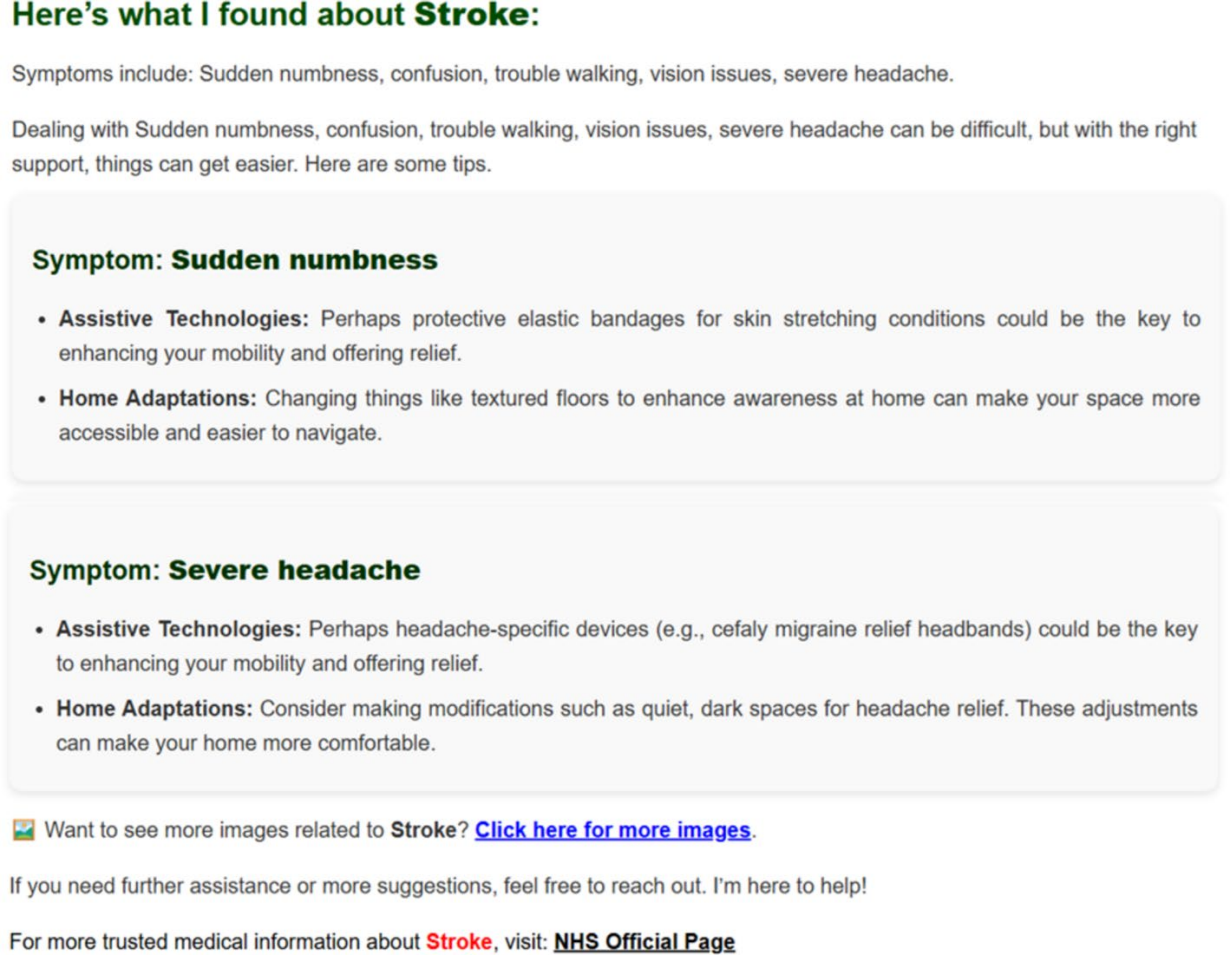
Textual and image-based personalised recommendations for stroke

This approach combines multiple advanced NLP techniques to achieve high match accuracy, effectively handling spelling errors, synonyms, and non-standard query phrasing. It is fully deployed on Hugging Face Spaces, offering scalable, real-time access through a user-friendly web interface. The GUI supports multi-tab interaction, enhancing usability and allowing for smooth navigation across different tools. However, the large codebase increases maintenance requirements, and performance can vary when dealing with vague or mixed-intent queries. The system also depends on pre-processed datasets, which may require periodic manual updates. Additionally, response times can be slower due to limitations of the free deployment tier, which restricts server resources and concurrent usage. The output, as shown in the Fig. 14, consists of conversational, personalised suggestions based on either disease or symptom input, including dynamically generated recommendations for assistive devices and home adaptations, all delivered through a modern, accessible web interface. This multitiered architecture represents the final deployed system from this research study, achieving a query matching accuracy (QMA) of 98%. QMA measures the proportion of user queries for which the system correctly retrieves and ranks the clinically and functionally appropriate assistive technology and housing adaptation recommendations, reflecting end-to-end retrieval performance rather than disease-label classification accuracy.

The progressive development of the proposed AI-driven framework, from a limited single-condition dataset to a flexible, symptom-driven conversational system, is examined in this study. Dataset-1 highlights the limitations of narrowly scoped, disease-specific data, resulting in low predictive accuracy. Dataset-2 broadens the scope to five conditions and multiple machine learning models, yielding moderate improvements while remaining constrained by static disease labels. Finally, Dataset-3 introduces a scalable and personalised framework powered by large language models and real-time web interfaces, enabling natural language input and context-aware, empathetic recommendations. This progression demonstrates a clear shift from rule-based systems to dynamic, AI-enhanced applications capable of handling complex, user-driven queries.

This evaluation is explicitly framed as a comparative feasibility and paradigm-suitability analysis, rather than as an exercise in performance optimisation or clinical benchmarking. Table 4 provides a comparative summary of the datasets, methodologies, input types, and key outcomes, highlighting the methodological progression across AI paradigms and the limitations observed at each stage that motivated the transition from disease-labelled learning to symptom-driven and LLM-based approaches. The emphasis is therefore on comparative insight and paradigm suitability, rather than on the individual systems as standalone contributions. Clinical validation with occupational therapists, local authorities, and end-users is beyond the scope of this study and is identified as future work.

**Table 4:** Summary of datasets, methods, and key outcomes

Dataset	Focus	Methods & models	User input type	Accuracy outcome	Key insights
Dataset-1	Single Disease (Muscular Dystrophy)	Fuzzy Matching + Decision Tree	Structured input (symptom-based)	< 20% Accuracy	Extremely limited data led to poor predictive performance. Model failed to capture emotional/behavioral complexity
Dataset-2	Five Diseases (Arthritis, Multiple Sclerosis, Parkinson’s disease, Muscular Dystrophy, and Stroke)	SMOTE-XGBoost, Random Forest, Parallel XGBoost, Stacked Ensemble, Voting Classifier	Structured (disease/symptom with categories)	12.80–26.24% Accuracy	Ensemble and XGBoost models improved prediction but lacked real adaptability. Limited by static disease labels
Dataset-3	Multiple Diseases & Symptoms	TF-IDF + Cosine Similarity, Fuzzy Matching, LLMs (DistilGPT2, FLAN-T5), Synonym Mapping, Conversational Interfaces	Structured, unstructured, or natural language input	Highly Flexible, Conversational, Personalised Responses (Qualitative Success)	Allowed symptom or disease based interactive query input. Context-aware LLM models improved relevance and empathy. Most accurate and user-friendly. Enabled real-time, multi-modal interaction

## Conclusions and future enhancements

The system developed uses a combination of NLP techniques and ML models to offer intelligent and personalised recommendations for assistive technologies and housing adaptations. The core of the system is powered by large language models such as FLAN-T5, which generate dynamic, context-aware responses tailored to user queries. The data used in the system is pre-processed from multiple Excel sheets containing information on diseases, symptoms, assistive devices, and housing factors as provided in the appendixes. Fuzzy matching techniques via the fuzzy-wuzzy library were employed to identify the best matches between user inputs and the dataset, allowing the system to handle various phrasings and synonyms. To further enhance flexibility, the system uses a synonym map to expand its recognition of related terms. Built with FastAPI, the web interface allows users to input queries related to diseases and symptoms, receiving customised suggestions. The system leverages a comprehensive dataset sourced from reputable institutions like the NHS and Mayo Clinic, incorporating over 500 types of diseases. This extensive dataset enhances the coverage and diversity of the recommendations provided. By integrating a wide range of diseases, symptoms, and their associated assistive technologies and housing adaptations, the system can offer more precise and personalised insights, making it a valuable tool for users with various medical conditions.

A key area for enhancement is the expansion and diversification of the underlying datasets, including larger and more comprehensive resources with multilingual disease descriptions, culturally diverse home adaptation needs, and condition-specific assistive technology mappings, which will improve both the accuracy and inclusivity of the system. The integration of data from open healthcare standards such as SNOMED CT, ICF, and ICD-11, together with real-world patient case studies and structured clinical records, will further enrich the semantic network used for matching and strengthen the system’s ability to address rare conditions and nuanced use cases. Alongside dataset growth, future work will extend this standards-aligned feasibility framework through human-centred design, including formal validation with occupational therapists and local authorities, small-scale user studies in real housing contexts, and the integration of real-time user feedback and active learning to support clinically, technically, and operationally deployable decision support.

## Electronic Supplementary Material

Below is the link to the electronic supplementary material.

Appendix 1

Appendix 2

Appendix 3, 4 and 5

## Data Availability

The authors confirm that the data supporting the findings of this study are based on publicly available sources and are cited within the manuscript. The data can be accessed via the URL below: [https://www.who.int/standards/classifications/international-classification-of-functioning-disability-and-health] [https://www.iso.org/standard/72464.html] [https://www.mayoclinic.org/diseases-conditions] [https://www.nhsinform.scot/illnesses-and-conditions/a-to-z/].

## References

[1] Abdi S, Spann A, Borilovic J, De Witte L, Hawley M. Understanding the care and support needs of older people: a scoping review and categorisation using the WHO international classification of functioning, disability and health framework (ICF). BMC Geriatr. 2019;19(1):195. 10.1186/s12877-019-1189-9. 31331279 10.1186/s12877-019-1189-9 PMC6647108

[2] Aclan R, George S, Block H, Lane R, Laver K. Middle aged and older adult’s perspectives of their own home environment: a review of qualitative studies and meta-synthesis. BMC Geriatr. 2023;23(1):707. 10.1186/s12877-023-04279-1. 37907851 10.1186/s12877-023-04279-1 PMC10619279

[3] Adams S, Hodges C. Home adaptations for disabled people: A detailed guide to related legislation, guidance and good practice. Foundations. 2018. https://www.housinglin.org.uk/_assets/Resources/Housing/Support_materials/Reports/HLIN_CaseStudyReport_Home-Adaptations.pdf

[4] Borchani H, Varando G, Bielza C, Larrañaga P. A survey on multi-output regression. WIREs Data Min Knowl Discov. 2015;5:216–33. 10.1002/widm.1157.

[5] Bottery S, Varrow M, Thorlby R, Wellings D. A fork in the road: Next steps for social care funding reform. The King’s Fund. 2018. https://www.kingsfund.org.uk/publications/fork-road-social-care-funding-reform

[6] Breiman L. Random Forests. Mach Learn. 2001;45:5–32. 10.1023/A:1010933404324.

[7] Carnemolla P, Bridge C. A scoping review of home modification interventions—mapping the evidence base. Indoor Built Environ. 2020;29(3):299–310. 10.1177/1420326X18761112.

[8] CDC. Disability and Health Overview: Impairments, Activity Limitations, and Participation Restrictions, Disability and Health Overview | CDC. 2024.

[9] Centre for Ageing Better (2020). Adapting for ageing: Good practice and innovation in home adaptations. https://ageing-better.org.uk/resources/adapting-for-ageing. Accessed on 18 March 2026.

[10] Chawla NV, Bowyer KW, Hall LO, Kegelmeyer WP. SMOTE: synthetic minority over-sampling technique. J Artif Intell Res. 2002;16:321–57. 10.1613/jair.953.

[11] Chen T, Guestrin C. XGBoost: a scalable tree boosting system. In: Proceedings of the 22nd ACM SIGKDD international conference on knowledge discovery and data mining. 2016. pp. 785–794. 10.1145/2939672.2939785

[12] Department of Health and Social Care. Assistive technology research and development work: 2023 to 2024. 2025. https://www.gov.uk/government/publications/assistive-technology-research-and-development-work-2023-to-2024

[13] Foundations UK. National healthy housing awards 2024: Winners revealed. 2024. https://www.foundations.uk.com/national-healthy-housing-awards-2024-winners-revealed

[14] Greenhalgh T, Wherton J, Sugarhood P, Hinder S, Procter R, Stones R. What matters to older people with assisted living needs? A phenomenological analysis of the use and non-use of telehealth and telecare. Soc Sci Med. 2013;93:86–94. 10.1016/j.socscimed.2013.05.036. 23906125 10.1016/j.socscimed.2013.05.036

[15] Habinteg Housing Association. Written evidence to the House of Lords—Housing and built environment. 2021. https://committees.parliament.uk/writtenevidence/124998/html

[16] Hender JM, Rodgers TL, Dean DL, Nunamaker JF. Improving group creativity: Brainstorming versus non-brainstorming techniques in a GSS environment. In: Proceedings of the 34th annual Hawaii international conference on system sciences. 2001. p. 10. 10.1109/HICSS.2001.926248

[17] Heywood F. The health outcomes of housing adaptations. Disabil Soc. 2004;19(2):129–43. 10.1080/0968759042000181767.

[18] Heywood F. Understanding needs: a starting point for quality. Hous Stud. 2004;19(5):709–26. 10.1080/0267303042000249161.

[19] Housing Grants, Construction and Regeneration Act 1996, c. 53, s. 34 (UK). Online. https://www.legislation.gov.uk/ukpga/1996/53/contents. Assessed on 10 Sept 2025.

[20] Imrie R. Universalism, universal design and equitable access to the built environment. Disabil Rehabil. 2012;34(10):873–82. 10.3109/09638288.2011.624250. 22054109 10.3109/09638288.2011.624250

[21] Institute of Development Studies. What is a “development” research project? Transforming ideas of development through development research. 2025. https://www.ids.ac.uk/opinions/what-is-a-development-research-project-transforming-ideas-of-development-through-development-research/

[22] ISO. Assistive products—Classification and terminology. 2022. https://www.iso.org/standard/72464.html

[23] Kasanen E, Siitonen A. The Constructive Approach in Management Accounting Research. Journal of ManagementAccounting Research. 1993;5.

[24] Kuncheva LI. Combining pattern classifiers: methods and algorithms. 1st ed. Wiley; 2004. 10.1002/0471660264

[25] Lan F. Research on text similarity measurement hybrid algorithm with term semantic information and TF-IDF method. Adv Multimed. 2022;2022:1–11. 10.1155/2022/7923262.

[26] Mackintosh S, Leather P. The Disabled Facilities Grant: Before and after the introduction of the Better Care Fund. Foundations. 2016. https://www.foundations.uk.com/policy_reports/the-disabled-facilities-grant-before-and-after-the-introduction-of-the-better-care-fund/

[27] Mayo Clinic. Diseases and conditions. 2019. https://www.mayoclinic.org/diseases-conditions

[28] Muscular Dystrophy UK. (n.d.). Adaptations manual for children and adults with muscle-wasting conditions. https://www.musculardystrophyuk.org

[29] NHS Inform. (n.d.). A to Z list of common illnesses and conditions. https://www.nhsinform.scot/illnesses-and-conditions/a-to-z/

[30] NHS Digital. Health Survey for England 2021: Part 2—Adults’ Health. 2023. https://digital.nhs.uk/data-and-information/publications/statistical/health-survey-for-england/2021-part-2/adult-health-general-health

[31] Office for Health Improvement and Disparities (OHID). Falls: applying All Our Health. 2021. https://www.gov.uk/government/publications/falls-applying-all-our-health/falls-applying-all-our-health?utm_source=chatgpt.com. Accessed 18 Jan 2026.

[32] Oyegoke AS, Ajayi S, Abbas MA, Ogunlana S. Development of a smart home suitability indicator and indicative self-assessment platform for the disabled facilities grants (DFGs). Int J Build Pathol Adapt. 2025;43(2):139–58. 10.1108/IJBPA-03-2023-0026.

[33] Royal College of Occupational Therapists. Care homes and equipment guiding principles for assessment and provision; 2019a

[34] Royal College of Occupational Therapists. Adaptations without delay: A guide to planning and delivering home adaptations differently; 2019b

[35] Saleela D, Oyegoke AS, Dauda JA, Ajayi SO. Development of AI-driven decision support system for personalized housing adaptations and assistive technology. J Aging Env. 2025. 10.1080/26892618.2025.2534956. 10.1007/s44163-026-01130-5 PMC13453988 42577299

[36] SenseOrg. Complex disabilities in the UK. 2022. https://www.sense.org.uk/about-us/research/complex-disabilities-overview/

[37] Sixsmith J, Sixsmith A, Fänge AM, Naumann D, Kucsera C, Tomsone S, et al. Healthy ageing and home: the perspectives of very old people in five European countries. Soc Sci Med. 2014;106:1–9. 10.1016/j.socscimed.2014.01.006. 24524960 10.1016/j.socscimed.2014.01.006

[38] Thomson H, Thomas S, Sellstrom E, Petticrew M. Housing improvements for health and associated socio-economic outcomes. Cochrane Database Syst Rev. 2013. 10.1002/14651858.CD008657.pub2. 10.1002/14651858.CD008657.pub2 PMC12551615 23450585

[39] Torres GG, Valadão YDN, Cruz TR, Müller I. Assistive Technology for Fall Detection Development of Integrated Wearable Sensor to Smart Home System. In: Anais Estendidos Do XI Simpósio Brasileiro de Engenharia de Sistemas Computacionais (SBESC Estendido 2021), pp. 140–145. 2021. 10.5753/sbesc_estendido.2021.18506

[40] UK Government (2016). Statutory guidance on Access to and use of buildings: Approved Document M. Online. https://www.gov.uk/government/publications/access-to-and-use-of-buildings-approved-document-m. Assessed on 18 March 2026.

[41] Whitehead PJ, Golding‐Day MR. The lived experience of bathing adaptations in the homes of older adults and their carers (BATH‐OUT): a qualitative interview study. Health Soc Care Community. 2019;27(6):1534–43. 10.1111/hsc.12824. 31373420 10.1111/hsc.12824 PMC6851978

[42] Wittenberg R, Comas-Herrera A, Pickard L, Darton R, Davies B. Financing long-term care for elderly people. In: Paper presented at the British Society of Gerontology 29th Annual Conference, Oxford, United Kingdom. 2000. http://eprints.lse.ac.uk/id/eprint/61114

[43] Woolham J, Benton C. The costs and benefits of personal budgets for older people: evidence from a single local authority. Br J Soc Work. 2013;43(8):1472–91. 10.1093/bjsw/bcs086.

[44] World Health Organization. International classification of functioning, disability and health (ICF). WHO. 2001. https://www.who.int/publications/i/item/9789241564182

[45] Wu J, Grundy EM. Housing adaptations and older adults’ health trajectories by level of initial health: evidence from the English Longitudinal Study of Ageing. Age Ageing. 2025;54(2):afaf023. 10.1093/ageing/afaf023. 39957554 10.1093/ageing/afaf023 PMC11831028

[46] Zhang M-L, Zhou Z-H. A review on multi-label learning algorithms. IEEE Trans Knowl Data Eng. 2014;26(8):1819–37. 10.1109/TKDE.2013.39.

[47] Zhou W, Oyegoke AS, Sun M. Causes of delays during housing adaptation for healthy aging in the UK. Int J Environ Res Public Health. 2019;16(2):192. 10.3390/ijerph16020192. 30641889 10.3390/ijerph16020192 PMC6352386

